# YoeB–ribosome structure: a canonical RNase that requires the ribosome for its specific activity

**DOI:** 10.1093/nar/gkt742

**Published:** 2013-08-14

**Authors:** Shu Feng, Yun Chen, Katsuhiko Kamada, Han Wang, Kai Tang, Meitian Wang, Yong-Gui Gao

**Affiliations:** ^1^School of Biological Science, Nanyang Technological University, 637551 Singapore, ^2^RIKEN Advanced Science Institute, Saitama 351-0198, Japan, ^3^Swiss Light Source, Paul Scherrer Institut, CH-5232, Switzerland and ^4^Institute of Molecular and Cell Biology, A-STAR, 138673, Singapore

## Abstract

As a typical endoribonuclease, YoeB mediates cellular adaptation in diverse bacteria by degrading mRNAs on its activation. Although the catalytic core of YoeB is thought to be identical to well-studied nucleases, this enzyme specifically targets mRNA substrates that are associated with ribosomes *in vivo*. However, the molecular mechanism of mRNA recognition and cleavage by YoeB, and the requirement of ribosome for its optimal activity, largely remain elusive. Here, we report the structure of YoeB bound to 70S ribosome in pre-cleavage state, revealing that both the 30S and 50S subunits participate in YoeB binding. The mRNA is recognized by the catalytic core of YoeB, of which the general base/acid (Glu46/His83) are within hydrogen-bonding distance to their reaction atoms, demonstrating an active conformation of YoeB on ribosome. Also, the mRNA orientation involves the universally conserved A1493 and G530 of 16S rRNA. In addition, mass spectrometry data indicated that YoeB cleaves mRNA following the second position at the A-site codon, resulting in a final product with a 3′–phosphate at the newly formed 3′ end. Our results demonstrate a classical acid-base catalysis for YoeB-mediated RNA hydrolysis and provide insight into how the ribosome is essential for its specific activity.

## INTRODUCTION

Toxin–antitoxin (TA) systems were originally identified on low-copy number plasmids, where their function is to maintain the plasmid by post-segregational killing of plasmid-free cells. To date, TA systems have been found to be highly abundant in chromosomes of both Eubacteria and Archaea. One TA system is composed of two genes organised into an operon that encodes a stable toxin and its cognate, more labile antitoxin. Based on the nature and the action mode of the antitoxin, TA systems are grouped into five types (I–IV) (1,2). The toxins are always proteins, whereas the antitoxins are either RNAs (type I: antitoxin RNA binds to the toxin-encoding mRNA to suppress its translation; type III: antitoxin RNA binds to the toxin protein to interfere with its activity) or proteins (type II: antitoxin protein binds to the toxin to cause its inactivation; type IV: antitoxin protein competes with the toxin for the same binding target; type V: antitoxin protein directly cleaves the toxin-encoding mRNA). Of the five types of TA systems, type II was discovered first, and later revealed to be the most common (1). There are at least eight well-characterized type II TA systems in *Escherichia coli*, including ribosome–dependent RelE-RelB, YoeB-YefM, YafO-YafN, YafQ-DinJ, and ribosome–independent MazF-MazE, ChpBK-ChpBI, HipA-HipB and MqsR-MqsA. RelE and MazF have been extensively studied among the type II TA systems. RelE is an endoribonuclease that cleaves the mRNA codon in the A site in a ribosome-dependent manner (3). MazF is an ACA sequence-specific, ribsosome-independent single-stranded mRNA endoribonuclease (4,5). However, some data have suggested that MazF may work in a similar manner as RelE (6).

In the case of the type II TA systems, antitoxin and toxin generally form a tight complex which inhibits the activity of the toxin. In response to environmental stress, the labile antitoxins are prone to be digested by stress-induced proteases such as Lon or bacterial proteasome systems, enabling the free toxins to exert their functions, leading to cell growth inhibition and adaptation (7). The molecular targets of the type II TA systems are highly diverse, and deciphering these targets is tremendously important for a better understanding of their functions. Eventually, this has considerable potential for applications, for example, as new components of the genetic toolbox and as targets for novel antibacterial drugs (8,9). In particular, identifying the TA systems and their targets in bacterial pathogens, such as *Staphylococcus aureus*, has attracted intensive interest and effort in recent years due to the emergence of multidrug resistance in these pathogens.

**Figure 1.**
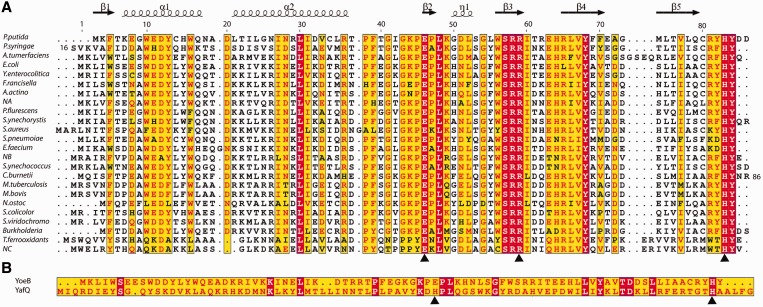
Alignments of YoeB homologues, as well as YoeB and YafQ. (**A**) Alignment of YoeB homologues. All proteins are named by their strains except for NA, NB and NC referring to the three predicted YoeB homologues in *Nitrosomonas europaea*. The secondary structure and sequence numbering are based on *E. coli* YoeB in this study. Three conserved residues Glu46 (general base), Arg59 (stabilization of transition state) and His83 (general acid), marked by black triangle, are required for YoeB activity. (**B**) Alignment of YoeB and YafQ. As a ribosome-dependent endonuclease, YafQ is another family of bacterial toxin. The general base and general acid in YoeB are glutamic acid and histidine (indicated by black triangle), which are universal in ribonuclease. Given that the pKa of histidine is close to neutral, thus making it the most effective candidate serving as general acid or base, His50 in YafQ could replace the role of Glu46 in YoeB to work as a general base, therefore, the mRNA cleavage mechanism by YafQ could be proposed to be similar as that by YoeB.

Among the characterized TA systems, YoeB-YefM is widespread among plasmids and genomes of Eubacteria and Archaea. Originally identified as Axe-Txe in a multidrug-resistant clinical isolate of *Enterococcus faecium* (10), YoeB-YefM has since been found in a large number of bacteria, including major pathogens such as *Staphylococcus aureus*, *Streptococcus pneumoniae*, *Mycobacterium tuberculosis* and *Yersinia enterocolitica* (Figure 1) (11). Recently, YoeB-YefM was identified as the first TA system in *Streptomyces* (12).

YoeB appears to be a homologue of the well-characterized RelE based on the structural similarity of the catalytic core. However, several differences can be noted. The two share only 15% sequence identity. In contrast to RelE, YoeB shows only partial activity in some biochemical assays (13). Additionally, the two interact with ribosome in distinct ways from each other. YoeB associates with the 50S after ribosome dissociation (14), and RelE interacts only the 16S rRNA (15). YoeB and RelE inhibit translation by affecting initiation or elongation, respectively (1). YoeB also preserves residues (Glu46 and His83) for general base/acid catalysis conserved in toxin YafQ (Figure 1) and in other RNases (11,13). These differences suggest that YoeB is a more typical mRNA endoribonuclease than RelE, but somehow it still requires the ribosome for its specific activity. Therefore, structural information on the complex of YoeB bound to ribosome is of considerable interest for a better understanding of the function and catalytic mechanism of TA type II toxins. This motivated us to determine the crystal structure of YoeB with the ribosome trapped in a state before mRNA cleavage. Together with the biochemical data showing the mRNA cleavage site following the second position of the A-site codon, our results offer the structural basis for a classical cleavage mechanism, which is common to many other toxins.

## MATERIALS AND METHODS

### Preparation of ribosomes, tRNA, mRNA and YoeB

*Thermus thermophilus* ribosomes were purified as described previously (16). *Escherichia coli* tRNA^fMet^ was purchased from Sigma. The mRNA MOO4b with the sequence 5′ GGCAAGGAGGUAAAAAUG**UAA**AAAA 3′ was synthesized with 2′-O-methylation modification for the three nucleotides at the A-site (bold) to trap the ribosome-YoeB complex in a pre-cleavage state.

*Escherichia coli* YeoB was cloned, expressed and purified using the same procedure as described previously (13). Briefly, YoeB and His-tagged YefM were co-expressed in *E. coli* BL21(DE3) strain, and YefM-free YoeB was eluted from a Ni^2+^ column to which YoeB-YefM complex was bound by a buffer containing 6M Guanidine-HCl. Denatured YoeB was then refolded by gradual dialysis against 25 mM HEPES (pH 7.5), 200 mM NaCl and 1 mM Dithiothreitol (DTT). Subsequently, the refolded YoeB was purified by Heparin-Sepharose and gel filtration chromatography and finally concentrated to 350 μM in dialysis buffer.

### Complex formation and crystallization

Complexes of YoeB with 70S ribosome, tRNA^fMet^ and mRNA (MOO4b) were crystallized as previously described (16). All complexes were formed in buffer G [5 mM HEPES (pH 7.5), 50 mM KCl, 10 mM NH_4_Cl, 10 mM Mg-acetate and 6 mM 2-mercaptoethanol]. 70S ribosomes at a final concentration of 4.4 μM were incubated with 8.8 μM mRNA and 13.2 μM tRNA^fMet^ at 55°C for 30 min. YoeB was added to a final concentration of 44 μM and incubated at 37°C for 30 min. The detergent Deoxy Big Chap was prepared as 14 mM in buffer G and was added to the complexes with a final concentration of 2.8 mM.

Crystals were grown via sitting-drop vapor diffusion method using 2.4 μl of complex sample plus 2 μl of reservoir solution containing 0.1 M Tris-HAc (pH 7.2), 0.2 M KSCN, 4.1–4.3% (w/v) Polyethylene glycol (PEG) 20K and 4.1–4.3% (w/v) PEG 550MME and left to equilibrate at 20°C. Crystals with stick morphology were grown to a full size of ∼1000 × 100 × 100 μm within 1 week. Crystals were equilibrated overnight by 10% increase of PEG, then transferred into mother solution plus 25% PEG550MME (stepwise increase) and frozen by plunging into liquid nitrogen.

### Data collection, refinement and model building

Crystals were screened on PXI beamline at the Swiss Light Source, and diffraction data were collected at 100K. All data were processed with X-ray Detector Software (XDS) (17). The empty 70S ribosome structure was used as an initial model (15), and refinement (including rigid body refinement of the initial model) was carried out with Crystallography and NMR System (CNS) (18). The difference density map clearly revealed the presence of the mRNA and tRNA ligands. All model building was done using COOT (19,20), and the electron density map was generated with CNS (18). The crystallographic data and refinement are summarized in Table 1. All figures were made with PyMOL (DeLano Scientific).

**Table 1.** Summary of crystallographic data and refinement statistics

Data collection	
Space group	P2_1_2_1_2_1_
Unit cell dimensions	
a,b,c (Å)	a = 211.6, b = 455.4, c = 616.9
α,β,γ (°)	α = β = γ = 90
Resolution (Å)	50–3.35 (3.4–3.35)^a^
R_merge _(%)	15.1 (106.2)
I/σI	9.3 (1.6)^b^
Completeness (%)	99.7 (99.8)
Redundancy	5.1 (4.7)
Refinement	
Resolution (Å)	50–3.35
No. of unique reflections	842970
R_work_/R_free_ (%)	22.1/26.1
No. of atoms	298211
RNA	199460
Protein	98040
Ions	711
Average B factor (Å^2^)	
RNA	111
Protein	125
Ions	92
R.m.s.deviations	
Bond length (Å)	0.006
Bond angle (°)	1.2

^a^Numbers in parentheses refer to outer resolution shell.

^b^I/σ = 2.0 at 3.4 Å.

### MALDI mass spectrometry

The programmed 70S ribosome (4.4 μM) formed in polymix buffer (20 mM HEPES pH 7.5, 95 mM K-glutamate, 5 mM Mg-acetate, 5 mM NH4Cl, 0.5 mM CaCl_2_, 1 mM Spermindine, 8 mM putrescine, and 1mM DTT) was incubated with the unmodified MOO4b mRNA (3.5 μM) in the presence or absence of 2.2 μM YoeB for 30 min at 37°C. The reaction products were isolated with phenol-chloroform extraction and ethanol precipitation, separated by 18% PAGE with 8 M urea. After gel purification and ethanol precipitation, the RNA fragments were dissolved in water. Before MALDI analysis, the samples were finally desalted and prepared with ZipTip C18 pipette tips (Millipore), and then analyzed with MALDI TOF/TOF ABI4800 in a positive ion, linear mode.

## RESULTS AND DISCUSSION

### Overall structure of YoeB bound to ribosome

**Figure 2.**
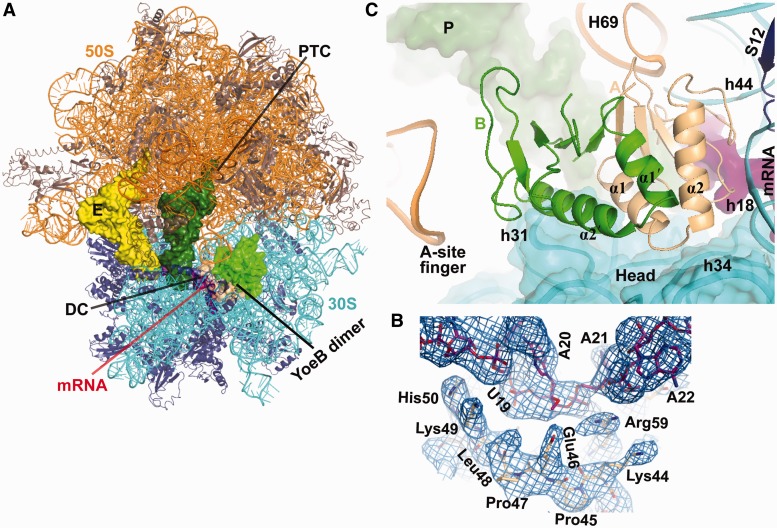
Structure of YoeB bound to the 70S ribosome. (**A**) Overall view of the complex with YoeB dimer (colored lightorange for monomer A and green for monomer B). The peptidyl transferase center (PTC) and the decoding center (DC) are indicated. (**B**) Unbiased difference Fourier electron density map displayed at 1.2σwith refined YoeB and mRNA. (**C**) Close-up of YoeB binding site. The N-terminal two helices for both YoeB monomers A and B are labeled. The 16S head is shown as cartoon with transparent surface. RNA helices are numbered with *E. coli* sequence, prefixed by H for 23S rRNA and h for 16S rRNA.

The crystal structure of *T. thermophilus* 70S ribosome in complex with YoeB, tRNA^fMet^ in the peptidyl (P) and exit (E) sites, and mRNA was refined to 3.35 Å resolution [I/(σI) = 2 at 3.4 Å] with a final R/R_free_ of 0.221/0.261 (Figure 2A and Table 1), representing a pre-cleavage state. The unbiased difference Fourier density shows both mRNA and YoeB, in which the well-ordered side chains were clearly distinguishable (Figure 2B). Unexpectedly, the structure reveals that YoeB binds to the ribosomal A site as a homodimer, which consequently blocks the access of both A-site tRNA and translational factors, such as release and elongation factors to ribosome (Figure 2A and C). The dimer interface does not directly interact with any ribosomal components. However, the entire dimer complements the shape of the ribosomal A site, suggesting that the binding of YoeB to ribosome could stabilize the dimer formation. The YoeB monomer located in proximity to the decoding center is referred to as A, and the other monomer is referred to as B. Monomer A spans the head and body of the 30S subunit, with the RNase fold β-sheet contacting the A-site mRNA. Monomer B occupies the space between the head of 30S subunit and the A-site finger of 23S rRNA (Figure 2C). On binding to ribosome, a number of rearrangements in monomer A are observed where various interactions are made (Supplementary Figure S1). In particular, the C-terminal tail of monomer A swings toward mRNA by ∼3 Å. In contrast, we see little conformational change in monomer B.

### Interactions of YoeB with ribosome

**Figure 3.**
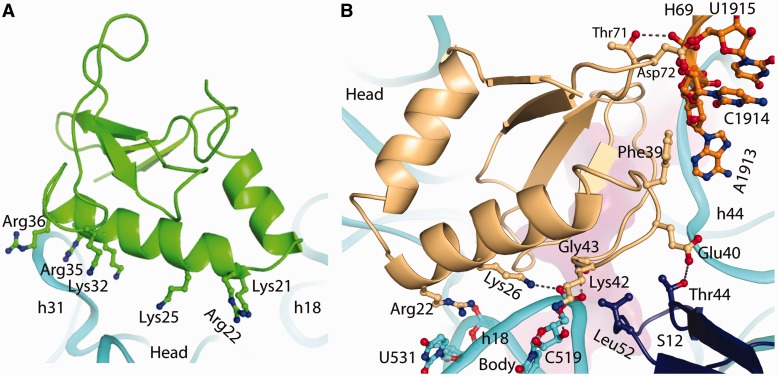
Interactions of YoeB with ribosome. (**A**) Interactions between monomer B of YoeB and helix 31 of 16S rRNA in the head. Monomer B, far apart form the ribosomal decoding center, with a large number of basic residues (shown as stick model) in helix α2 facing the 30S head. (**B**) Interactions of monomer A of YoeB with the body region of 30S (G530 and A1913 regions), H69 of 23S rRNA and ribosomal protein S12. The hydrogen-bonding interaction is indicated by dashed line.

Whether YoeB functions as a dimer *in vivo* is uncertain (13). Here, we describe the interactions of the two YoeB monomers with the ribosome. Monomer B, far apart form the ribosomal decoding center, has a large number of basic residues (residues 21, 22, 25, 32, 35 and 36) in the helix α2 facing the 30S head (Figure 3A). Of these residues, Lys32, Arg35 and Arg36 are within hydrogen-bonding distance to h31 of 16S rRNA in the 30S head. Except for these interactions, monomer B does not make direct contacts with other ribosomal components.

**Figure 4.**
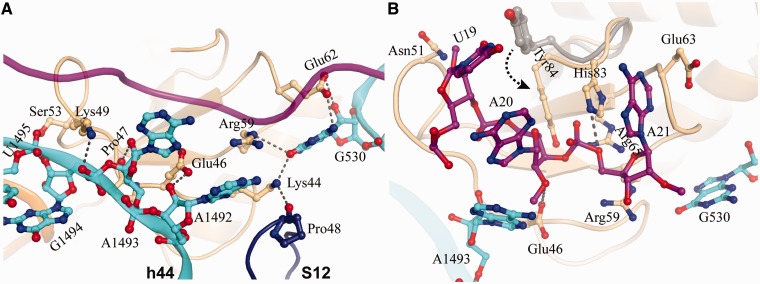
Interactions at the catalytic site (**A**) Interactions of YoeB with ribosome at the decoding center. The mRNA is shown as ribbon colored magenta. The residues in YoeB within hydrogen-bonding distance to ribosome are indicated by dashed lines. (**B**) Active site of YoeB surrounded by the A-site codon. Catalytic residues Glu46 and His83 are within hydrogen-bonding distance to their reaction atoms, indicated by dashed lines. Compared with isolated YoeB colored gray, conformational change of C-terminal tail of YoeB (particularly Tyr84) was shown by an arrow.

In contrast to monomer B, monomer A forms extensive contacts with rRNAs (both 16S and 23S), ribosomal protein S12 and mRNA (Figures 2C and 3B). The N-terminal α1 and α2 form a V-shaped arrangement wedging into the cleft between the head and body of 30S, where the basic residues Arg22 and Lys26 interact with U531 and C519 in the decoding region (Figures 2C and 3B). On the opposite side of monomer A, it forms strong interactions with the tip of H69 of 23S rRNA, A1492/A1493 in h44 of 16S rRNA, and S12 (Figures 3B and 4A). The residues Thr71 and Asp72, located in the β-strand loop, establish hydrogen-bonding interactions with U1915 and C1914 in H69. Additionally, Phe39 enhances the interaction of YoeB with H69 via hydrophobic contact (A1913). H69, a highly conserved stem-loop (1906–1924) involved in the formation of intersubunit bridge B2a, is capable of interacting with translational factors such as release factors RF1 and RF2 (21,22), indicating a pivotal role in translation (23). YoeB tightly contacts H69, thereby likely interfering with its function. The neighbouring residue Glu40 of YoeB interacts with Thr44 located at the highly conserved β-loop of S12, which projects into the decoding center to participate in codon–anticodon recognition (24). In proximity, Lys42 forms bilateral contacts with ribose O3 of C519 in h18 of 16S rRNA and Leu52 in the β-loop of S12, via hydrogen bond and side-chain hydrophobic interaction, respectively (Figure 3B). Moreover, the main chain N atom of Gly43 contacts phosphate oxygen of C519 by ∼3.0 Å. In particular, a network of interactions involving YoeB, G530, S12, and mRNA is formed, of which Lys44 in YoeB interacts with the highly conserved Pro48 in S12 (Figure 4A). Pro48 is crucial for ribosome function, as mutation of the equivalent residue in *E. coli* S12 resulted in severe dominant growth defects (25).

At the decoding center, the loop linking α2 and β2 in YoeB also forms several tight interactions with the A1492 region of 30S, causing a significant conformational change in A1492 and A1493 (Figure 4A and Supplementary Figure S2). The conformationally flexible ‘decoding bases’ A1492 and A1493 are pulled completely out of h44 of 16S, which is complemented by a shift of the main chain toward the helical axis. As a result, a unique conformation for the ‘decoding bases’ is observed. Such a conformation is distinct from that observed in ribosome bound to RelE (15). In the 70S-RelE complex (15), A1493 stacks with A1913 of 23S rRNA, both A1493 and A1492 conformations are incompatible with the binding of YoeB.

Although the YoeB monomer A and RelE occupy a similar position at the ribosomal A site, their interactions with the ribosome are notably different (Supplementary Figure S3). RelE contacts almost exclusively 16S rRNA and shows only moderate hydrogen-bonding interactions with the decoding center. In contrast, YoeB makes extensive contacts with 16S rRNA, 23S rRNA and ribosomal protein S12. In particular, the region between residue 34 and 56 forms interactions with the decoding site involving the three universally conserved nucleotides G530, A1492 and A1493, which play a vital role in proper decoding by interacting with the codon–anticodon helix to construct the structural constraints necessary to discriminate the accuracy of base pairing (24,26). Moreover, YoeB strongly interacts with H69 of 23S rRNA, which is involved in the formation of an essential intersubunit bridge B2a, as well as implicated in termination by interacting with release factors (26). Despite the fact that these interactions may change after mRNA cleavage or during ribosomal subunit dissociation, together, our observations provide support for two previously reported features of YoeB. First, YoeB effectively inhibits protein synthesis by binding to ribosome and blocking the A site. Second, YoeB was found to associate with 50S subunit after 70S ribosome dissociation (14).

### The mRNA recognition by YoeB involving the ribosome

Despite YoeB binding to the ribosome as a dimer under our experimental conditions, monomer B is absolutely inaccessible to mRNA, demonstrating only one active site in YoeB dimer. The ribose O2 of the first nucleotide (U19) of the A-site codon interacts with Asn51 of YoeB (Figure 4B), appearing to prevent it from rotating back to contact P-site tRNA, in a similar way as observed in the RelE-bound state (15). The second and third nucleotides (A20 and A21) form direct interactions with a number of residues in YoeB, including Glu46, Arg59, Glu63, Arg65, His83 and Tyr 84, which are located in the β-sheet and the C-terminal loop (Figure 4B). These residues are highly conserved within the YoeB family (Figure 1), and most of them have been demonstrated to be indispensable for the activity of YoeB (11). As the result of binding to ribosome, conformational change in the YoeB C-terminal tail brings the two activity-required residues, His83 and Tyr84, into the present position, where tight interactions with A20 and A21 are made (Figure 4B). Interestingly, His83 and Glu63 hold the base of A21 by stacking to both sides. The universally conserved A1493 and G530 orient the mRNA substrate for subsequent cleavage, which is supported by additional interactions mediated by residues of YoeB. A1493 forms direct contacts with the second nucleotide A20, and G530 interacts with guanidinium group of Arg59, which subsequently contacts A21 at position 3 and the linked phosphate in A22.

The overall path of the mRNA in the presence of YoeB deviates remarkably from that seen in the ribosome with a cognate A-site tRNA (16) (Supplementary Figure S4), even though the nucleotides downstream of the A-site codon occupy the general mRNA channel in the 30S subunit (27). Surprisingly, the deformed A-site codons are in different conformations in the YoeB- and RelE-bound structures (Supplementary Figure S4), with distances between the phosphates of the A-site nucleotides 0.8, 6.5 and 4.6 Å, respectively. This is a consequence of the different interactions of YeoB and RelE with mRNA.

### YoeB cleaves mRNA following the second position of the A-site codon

**Figure 5.**
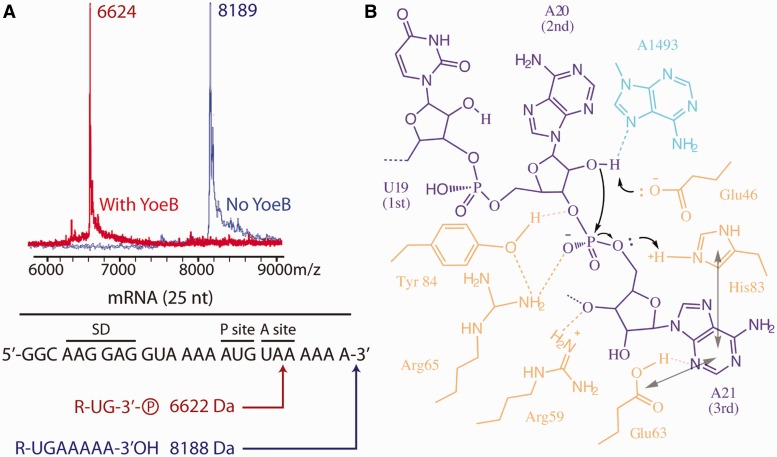
MALDI mass spectrometry analysis of cleaved mRNA fragment and a proposed mechanism. (**A**) Mass spectra and the corresponding RNA fragments. The position of cleavage site was deduced from the mass result. (**B**) Schematic presentation of the proposed mechanism for mRNA cleavage by YoeB (YoeB, mRNA and A1493 of 16S rRNA colored lightorange, magenta, cyan). The general base Glu46 deprotonates the 2′OH of A20, activating it for a nucleophilic attack on the phosphate following the second A-site nucleotide. The transition state is stabilized by Arg65, and Tyr84 is also involved in orienting mRNA and stabilizing the transition state. His83 acts as generate acid to protonate the 5′OH leaving group, and together with an exogenous water molecule to involve subsequent hydrolysis of 2′-3′ cyclic phosphate to the final RNA fragment with 3′-phosphate and 2′-OH. Stacking of the third base with both His83 and Glu63, and interaction of Arg65 with A21 facilitate the reorientation of mRNA for an inline attack.

To determine the exact cleavage site, analysis of the cleaved RNA fragment by mass spectrometry was carried out. When mRNA was incubated with the ribosome in the absence of YoeB, a mass of 8189 Da peak corresponding to the full-length mRNA was observed, whereas an RNA fragment with a mass of 6624 Da was detected in the presence of YoeB, implying that the cleavage occurred after the second position in the A-site codon (Figure 5A). Moreover, YoeB produces a final product with a 3′-phosphate at the newly formed 3′ end, unlike the final product with 2′–3′ cyclic phosphate at the 3′ end observed with RelE (15). As methylation of A-site mRNA nucleotides at 2′–O position completely abolished mRNA cleavage by YoeB (data not shown), a cleavage mechanism involving the 2′–OH induced hydrolysis was revealed. This is also the case with RelE (15), as well as other structurally related nucleases RNase T1 (28), RNase Sa (29) and RNase Sa2 (30). Notably, YoeB has been shown to cleave *in vivo* mRNA with diverse cleavage sites (12,14,31). Compared with the *in vitro* data here, the difference probably results from that *in vivo* the mRNA most likely shifts inside the ribosome allowing cleavage in diverse places, in a similar way as observed for RelE (15).

### A classical mechanism for the ribosome–dependent mRNA cleavage by YoeB

Based on our results and the previous mutation data (13), we propose a mechanism of mRNA cleavage by YoeB (Figure 5B), which resembles the mechanism accepted for RNase T1/RNase Sa (28,29). Namely, Glu46 acts as a general base, the negatively charged side chain deprotonates the ribose 2′–O of A20, which is thereby activated for an inline nucleophilic attack on the adjacent 3′–phosphate. The negatively charged trigonal bipyramidal transition state is likely stabilized by His83, which is proposed to donate a proton leading to the formation of the leaving 5′OH group during the final stage of catalysis. His83 thus acts as a general acid. The positively charged side chain of Arg65 is in position to stabilize the transition state, consistent with its necessity for the enzymatic activity reported previously (13). Subsequent hydrolysis of the resulting 2′–3′ cyclic phosphate to the final RNA fragment with 3′–phosphate and 2′–OH, involves an exogenous water molecule and His83, in a similar way as with RNase T1 (28). The conserved residue Tyr84 in the proximity of the scissile phosphodiester bond is involved in catalysis by orienting mRNA via its benzene ring and forming a network of hydrogen-bonding interaction with Arg65 and phosphate-oxygen at the cleavage site in mRNA. This allows us to rationalize mutation data on Y84A and Y84F, in which Y84A results in completely abolishing the activity, and Y84F shows compromised activity (13).

*In vivo*, YoeB-mediated mRNA cleavage occurs only within translated regions of the RNA in a ribosome-dependent manner (32). As described earlier in the text, the universally conserved A1493 and G530 are involved in mediating YoeB interactions with the substrate mRNA (Figure 4). Particularly, the direct contact of A1493 in the 16S rRNA with the base of mRNA A20, as well as the network of interactions between A1493, ribose O2 in A20 (site of hydrolysis), and carboxylic acid group of Glu46 (general base) rationalize the requirement of the ribosome for the activity of YoeB. Considering that the N7 atom of A1493 is in place to interact with ribose O2 in A20 and its potential capability for deprotonating, it is likely that A1493 might function as general base in the absence of Glu46, thereby explaining the compromised activity of Glu46 mutation (13). On the other hand, YoeB has a weak activity on naked mRNA, whereas RelE does not. YoeB retains a complete RNase fold with catalytic residues glutamic acid and histidine that have pKa values well-suited to act as general base/acid so that YoeB by itself may be capable of cleaving certain mRNA to some extent (13). The main contribution of the ribosome may therefore be to stabilize mRNA in a conformation suitable for attack by YoeB (12–14).

Based on our structure, the position of the first A-site codon is compatible with accommodating any base, and the second and third appear to favor adenine but can be replaceable by guanine. This is different from RelE, which shows a more strict sequence specificity toward A-site mRNA (6,33). Although cleavage by RelE was observed to be more frequent within the first ∼100 codons *in vivo* (34), YoeB has less requirement for the mRNA codon leading to its cleavage occurring immediately adjacent to the translational initiation site (14). Such distinct specificity of these two toxins may account for their different inhibition mechanisms. Namely, as proposed previously, YoeB acts as an inhibitor for translation initiation, whereas RelE inhibits elongation (1).

## CONCLUSION

It was proposed that ribosome-associated endoribonucleases in bacteria act as ‘adaptation enzymes’, each appropriate for a specific environmental condition (15). These ‘enzymes’ degrade mRNA in response to a range of conditions, such as environmental stress, viral infections and ribosome stalling, thereby modulating protein translation to reduce energy cost. RelE, the structurally related endoribonuclease of YoeB as well as a rare protein in bacteria, lacks the conserved catalytic residues (histidine and glutamate) and uses a non-canonical cleavage mechanism (15). In contrast, the widespread YoeB is a typical endoribonuclease. In this study, we determined the structure of YoeB with ribosome trapped in pre-cleavage state, which allows insights into the common and variable features of ribosome-dependent nucleases. Although YoeB executes a classical acid–base catalysis mechanism common to many RNases, its activity intimately depends on the interactions with the ribosome and mRNA as visualized here. These results provide valuable implications in understanding the molecular mechanism of YoeB family members and other ribosome-dependent endoribonucleases in translation regulation by mRNA degradation. Complementary approaches will be required to further improve our understanding of the intricate substrate requirements for this uncommon class of enzymes.

## ACCESSION NUMBERS

Atomic coordinates and structure factors have been deposited in Protein Data Bank (PDB) with accession codes 4byb, 4byc, 4byd and 4bye.

### SUPPLEMENTARY DATA

Supplementary Data are available at NAR Online, including [35–36].

## Supplementary Material

Supplementary Data
