# Supplementary material for: YoeB–ribosome structure: a canonical RNase that requires the ribosome for its specific activity
Source: Nucleic Acids Res. 2013 Aug 14;41(20):9549–56. doi: 10.1093/nar/gkt742 (PMC3814384; doi:10.1093/nar/gkt742)
Supplement: Supplementary Data [file supp_41_20_9549__index.html]

YoeB–ribosome structure: a canonical RNase that requires the ribosome for its specific activity — YoeB–ribosome structure: a canonical RNase that requires the ribosome for its specific activity — Supplementary Data 

# YoeB–ribosome structure: a canonical RNase that requires the ribosome for its specific activity

## Supplementary Data

files

**Files in this Data Supplement:**

- Supplementary Data - pdf file
