# Supplementary material for: YoeB–ribosome structure: a canonical RNase that requires the ribosome for its specific activity
Source: Nucleic Acids Res. 2013 Aug 14;41(20):9549–56. doi: 10.1093/nar/gkt742 (PMC3814384; doi:10.1093/nar/gkt742)
Supplement: Supplementary Data [file supp_gkt742_nar-01777-r-2013-File007.pdf]

Supplementary Data

YoeB–ribosome structure: A canonical RNase that requires the ribosome for its specific activity

Shu Feng[1]*, Yun Chen[1]*, Katsuhiko Kamada[3], Han Wang[1], Kai Tang[1],

Meitian Wang[4], Yong-Gui Gao[1,2]

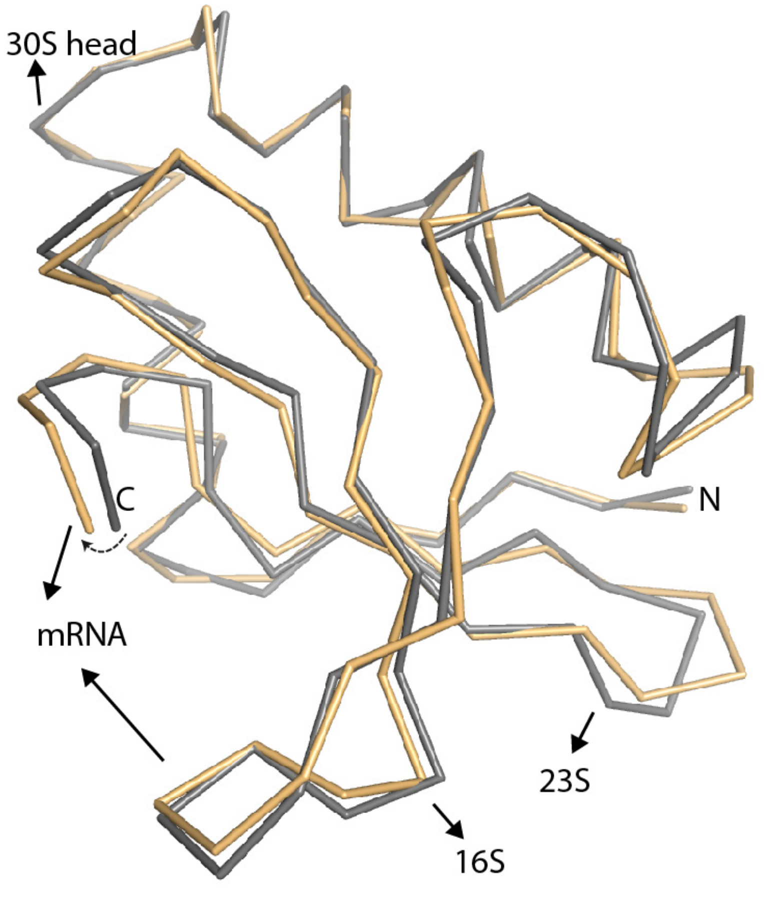

Supplementary Figure 1. Structural comparison of isolated YoeB (molecule A, grey) (13) with the ribosome-bound form (monomer A, lightorange). Upon binding to ribosome, the C-terminal of YoeB shifts towards mRNA, demonstrated by an arrow with dashed line. The regions contacting ribosome and mRNA are marked.

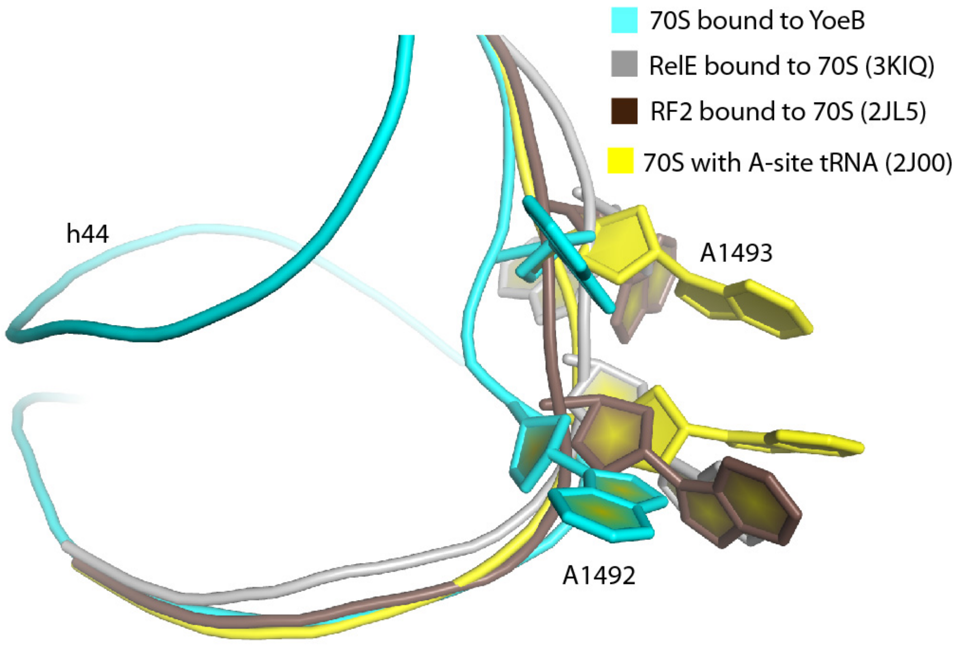

Supplementary Figure 2. Structural comparison of the decoding site A1492 and A1493. Conformations of the two universally conserved A1492 and A1493 at the decoding center in ribosome complexes including YoeB bound to 70S (cyan, this study), RelE bound to 70S (PDB: 3KIQ) (15), release factor 2 (RF2) bound to 70S (PDB: 2JL5) (22), and 70S with a cognate A-site tRNA (PDB: 2J00) (16) are presented. The conformation of A1492 and A1493 in the 70S complexed with elongation factor G (PDB: 2WRI) (35) is very similar to that of 2J00 (colored yellow here), thereby not shown in the figure. These two bases, stacking each other, are buried in the h44 of 16S rRNA in the empty ribosome structure (PDB: 2AVY) (36), also not shown here.

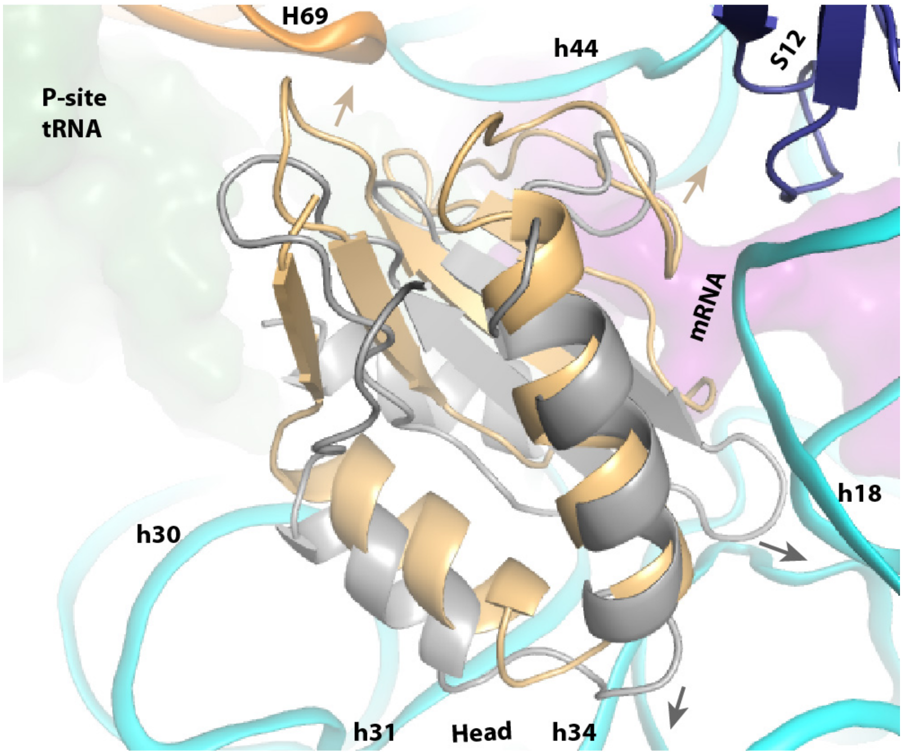

Supplementary Figure 3. The positioning of YoeB and RelE in the ribosome. The ribosome-bound RelE in pre-cleavage state (3KIQ) (15) was superposed to YoeB in the present structure by fitting on 16S rRNA. YoeB and RelE (grey) are shown as cartoon. YoeB forms extensive contacts with H69 of 23S rRNA, h44 of 16S rRNA, and protein S12 at the decoding regions (indicated by yellow arrows), whereas RelE make more interactions with h31 and h34 in the 30S head, and h18 in the 30S body (indicated by grey arrows).

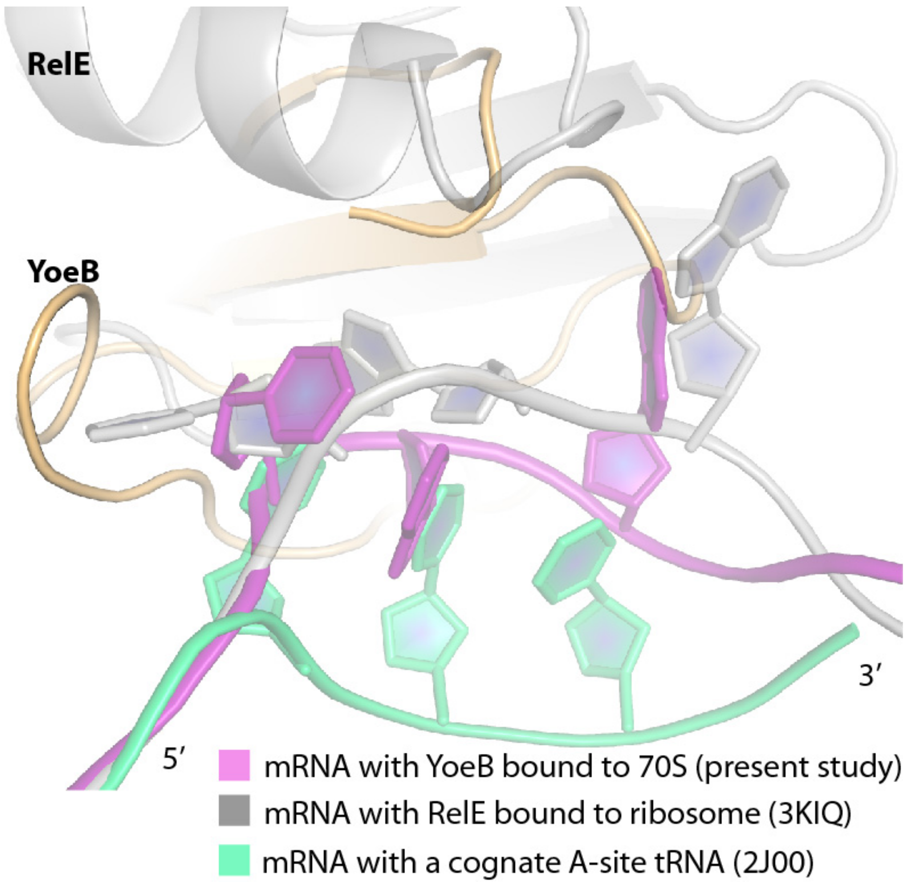

Supplementary Figure 4. Conformation of the A-site mRNA codon. The mRNAs in the presence of YoeB, RelE (15), and a cognate tRNA (16) are colored magenta, grey, and forest, respectively. The distances between the phosphates of the A-site nucleotides in the YoeB- and RelE-bound structures are 0.8, 6.5, and 4.6 Å, respectively.

REFERENCES

13. Kamada,K. and Hanaoka,F. (2005) Conformational change in the catalytic site of the ribonuclease YoeB toxin by YefM antitoxin. *Mol Cell* **19**, 497-509.

15. Neubauer,C., Gao,Y.G., Andersen,K.R., Dunham,C.M., Kelley,A.C., Hentschel,J., Gerdes,K., Ramakrishnan,V. and Brodersen,D.E. (2009) The

structural basis for mRNA recognition and cleavage by the ribosome-dependent endonuclease RelE. *Cell* **139**, 1084-1095.

16. Selmer,M., Dunham,C.M., Murphy,F.V.t., Weixlbaumer,A., Petry,S., Kelley,A.C., Weir,J.R. and Ramakrishnan,V. (2006) Structure of the 70S ribosome complexed with mRNA and tRNA *Science* **313**, 1935-1942.

22. Weixlbaumer,A., Jin,H., Neubauer,C., Voorhees,R.M., Petry,S., Kelley,A.C. and Ramakrishnan,V. (2008) Insights into translational termination from the structure of RF2 bound to the ribosome *Science* **322**, 953-956.

35. Gao,Y.G., Selmer,M., Dunham,C.M., Weixlbaumer,A., Kelley,A.C. and Ramakrishnan,V. (2009) The structure of the ribosome with elongation factor G trapped in the posttranslocational state. *Science* **326**, 694-699.

36. Schuwirth,B.S., Borovinskaya,M.A., Hau,C.W., Zhang,W., Vila-Sanjurjo,A., Holton,J.M. and Cate,J.H. (2005) Structures of the bacterial ribosome at 3.5 A resolution. *Science* **310**, 827-834.
